# Diarylheptanoids from *Alpinia officinarum* Cause Distinct but Overlapping Effects on the Translatome of B Lymphoblastoid Cells

**DOI:** 10.1155/2014/204797

**Published:** 2014-08-31

**Authors:** Tomohito Kakegawa, Saeko Takase, Eri Masubuchi, Ken Yasukawa

**Affiliations:** ^1^Faculty of Pharmaceutical Sciences, Josai International University, 1 Gumyo, Togane, Chiba 283-8555, Japan; ^2^School of Pharmacy, Nihon University, 7-7-1 Narashinodai, Funabashi, Chiba 274-8555, Japan

## Abstract

Diarylheptanoids (AO-0001, AO-0002, and AO-0003) isolated from *Alpinia officinarum* inhibit proinflammatory mediators and exhibit cytotoxic and antiviral activity. However, the precise mechanisms of action of these diarylheptanoids are unknown as are their effects on expression of specific genes. Here, we used a translatome analysis to investigate the mechanisms and modes of action of these three diarylheptanoids. Polysome-associated messenger RNAs (mRNAs) were prepared from diarylheptanoids-treated and control cells from a human B lymphoblastoid cell line; these mRNA samples were then used for microarray analysis. Microarray Data Analysis Tool version 3.2 was used to analyze the microarray data analysis; this software uses pathway information of the WikiPathways for gene ontology analysis. Each of the diarylheptanoids caused upregulation or downregulation of the same 37 and 286 genes, respectively. Among the 37 upregulated genes, 16 were related to mRNA processing based on the WikiPathways analysis. Our findings provided new insights into the mode of action of diarylheptanoids from *A. officinarum*.

## 1. Introduction

*Alpinia officinarum *belongs to the family Zingiberaceae and is known as lesser galangal.* A. officinarum* rhizomes have been used in many Asian cuisines and as traditional medicine; these rhizomes have been used as antiemetics, stomachics, and analgesics in Asia since ancient times. In a series of studies on bioactive compounds from natural sources, we found that a methanol extract from the rhizome of* A. officinarum *is effective in inhibiting 12-O-tetradecanoylphorbol-13-acetate- (TPA-) induced tumor promotion in skin of mice [1]. Diarylheptanoids isolated from* A. officinarum* have many reported effects; they inhibit the melanogenesis caused by B16 melanoma cells [2]; induce apoptosis, S-phase arrest, and differentiation of human neuroblastoma cells [3]; exhibit cytotoxic activity [4]; suppress inducible nitric oxide synthase expression [5]; inhibit biosynthesis of prostaglandin and leukotrienes [6, 7]; and inhibit proinflammatory mediators [8]. Additionally, diarylheptanoids reportedly have antiviral activity against influenza virus [9, 10], respiratory syncytial virus, poliovirus, measles virus, herpes simplex virus, and type 1 poliovirus [11, 12]. However, the precise mechanisms of action of these diarylheptanoids are undefined as are any effects they have on the expression of specific genes.

Over the last 10 years, translatome analyses of eukaryotic cells or tissues have been increasingly used by researchers. The polysome microarray approach, which was originally reported by Zong et al., is the most commonly used method for translatome analysis [13]. With this approach, mRNAs associated with several ribosomes (usually >3) are separated from mRNAs associated with fewer ribosomes; these polysome-associated mRNAs are then used to label probes on microarrays [14]. As genetic information transforms from DNA to protein, the cellular abundance of proteins is predominantly controlled at the level of translation [15]; observed correlations between mRNA levels and respective protein levels are low [16]. Analysis of the translatome, an intermediate level between the transcriptome and the proteome represented by polysome-associated mRNAs, has provided substantial and somewhat surprising new information [17].

In this study, we used this microarray-based approach to comprehensively identify the polysome-associated mRNAs in a human B lymphoblastoid cell line (BJAB) and to examine changes to this mRNA profile caused by each of the three* A. officinarum* diarylheptanoids.

## 2. Materials and Methods

### 2.1. Chemicals

**Figure 1:**
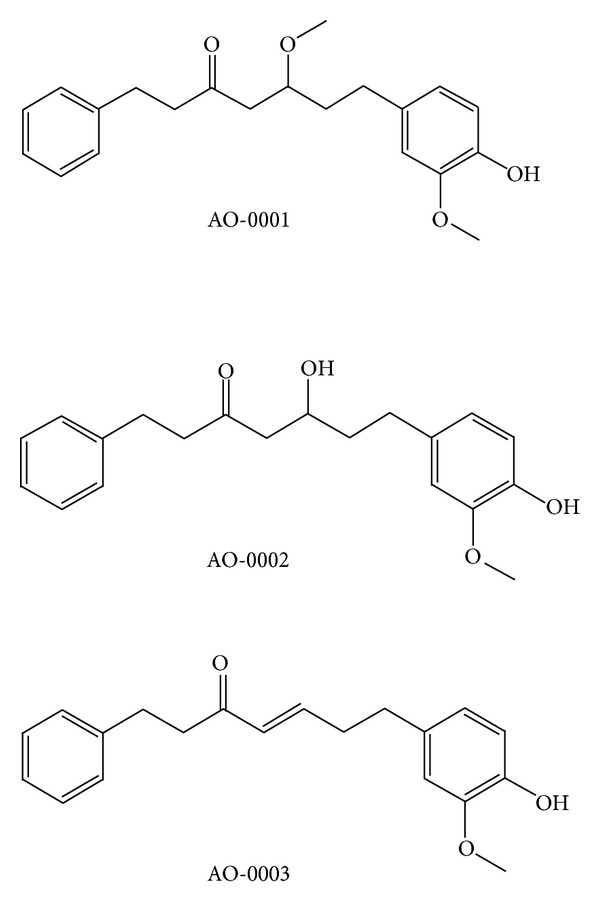
The chemical structures of AO-0001, AO-0002, and AO-0003.

Chemicals were purchased from Wako Pure Chemical Industries, Ltd., Osaka, Japan. AO-0001: (5*R*)-7-(4′′-hydroxy-3′′-methoxyphenyl)-5-methoxy-1-phenyl-3-heptanone, AO-0002: (5*R*)-5-hydroxy-7-(4′′-hydroxy-3′′-methoxyphenyl)-1-phenyl-3-heptanone, and AO-0003 : 7-(4′′-hydroxy-3′′-methxoyphenyl)-1-phenyl-4*E*-hepten-3-one (Figure 1) were isolated from the rhizome of* A. officinarum* as described previously [4]; each was stored as 40 mM stock solution in 100% dimethyl sulfoxide (DMSO) (final concentration of DMSO 0.1%).

### 2.2. Cell Culture

BJAB cells were grown in Roswell Park Memorial Institute (RPMI) 1640 medium (Sigma), 10% fetal bovine serum (Sigma), 5 *μ*g/mL amphotericin B (Bristol-Myers), and 10 *μ*g/mL gentamicin (Sigma). The cells were maintained at 37°C with 5% CO_2_ in a humid environment [18]. Proliferation of the BJAB B-lymphoblastoid cell line is rapidly and almost completely suppressed by picomolar concentrations of the immunosuppressive macrolide rapamycin [19]. This hypersensitivity to rapamycin of BJAB cells might indicate that the canonical translation system in BJAB is highly dependent on mTOR (mammalian target of rapamycin) and is highly activated. Thus, we used BJAB cells.

### 2.3. Cellular Fractionation and RNA Preparation

**Figure 2:**
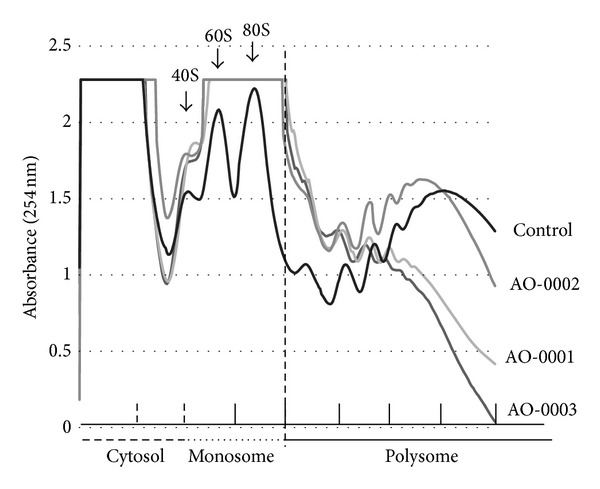
Polysome sedimentation profiles of fractions isolated from control BJAB cells and BJAB cells treated with AO-0001, AO-0002, or AO-0003. The cytosol fraction was layered onto a 0.5 M–1.5 M linear sucrose gradient (prepared by Gradient Master 107, Biocomp) in LSB (20 mM Tris-HCl pH 7.0, 10 mM NaCl, 3 mM MgCl_2_); the gradient tube was centrifuged at 47,000 rpm in a Hitachi SW55Ti rotor for 70 minutes. A Piston Gradient Fractionator (Biocomp) equipped with an absorbance monitor (254 nm) was used to separate each gradient into nine fractions (0.5 mL/fraction). The absorbance of each 9th fraction (0.5 mL) could not be measured because, in each case, the 9th fraction was retained at the bottom of the ultracentrifuge tube. Positions of ribosomal 40S and 60S subunits and 80S monosomes are indicated by arrows.

Polysome analysis was performed as described previously [18] with slight modifications. Briefly, 40 mL of exponentially growing BJAB cells (0.5 × 10^6^cells/mL) was untreated or was treated with DMSO-only, 40 *μ*M AO-0001, 40 *μ*M AO-0002, or 40 *μ*M AO-0003 for 3 hours. The concentration of each diarylheptanoid used for treatment was determined based on previous findings [2–5]. To prepare cytoplasmic extract for ribosomal fractionation, cells were washed with ice-cold RPMI 1640 medium containing 0.1 mg/mL cycloheximide and collected by centrifugation; each resulting pellet was homogenized with a Teflon pestle in an Eppendorf tube in ice-cold 0.375 mL of low salt buffer (LSB) (0.1 M NaCl, 3 mM MgCl_2_, 20 mM Tris-HCl [pH 7.6], and 1 mM dithiothreitol). Next, 100 *μ*L of lysis buffer (LSB with 0.2 M sucrose, 1.2% Triton N-101), 15 *μ*L of 5 M NaCl, and 50 *μ*L of 10 mg/mL heparin-Na in LSB were added to each homogenate; each mixture was centrifuged at 10,000 ×g for 5 min to clear the resultant supernatant of nuclei, mitochondria, and debris. Lysate (<0.4 mL) was layered over a 4.5 mL linear sucrose gradient solution (0.5–1.5 M in LSB, prepared by Gradient Master 107, Biocomp) in a 5 mL tube and centrifuged at 47,000 rpm for 70 minutes at 4°C in Hitachi RPS 55ST rotor. Each gradient was fractioned at a rate of 0.5 mL per minute by upward displacement via a Piston Gradient Fractionator (Biocomp), which was equipped with an absorbance monitor (BioLogic DuoFlow and BioLogic Optics Module II OM-1). Resulting profiles of absorbance at 254 nm are shown in Figure 2. Each fraction was stored at −30°C until use for total RNA extractions [18].

### 2.4. RNA Preparation for CodeLink Human Whole Genome Bioarray Analysis

The microarray analysis was performed as a trust analysis service at Filgen Incorporation (Nagoya, Japan). The Agilent Bioanalyzer 2100 RNA Nano LabChip (Agilent Technologies) analysis system was used to assess the RNA quality of each experimental sample. Under standard conditions, processing of RNAs used with the CodeLink Human Whole Genome Bioarray (Applied Microarrays, Inc.) was in accordance with methods described in the manufacturer's instructions, as subsequently detailed. MessageAmp II-Biotin Enhanced Kits (Ambion) were used to synthesize 10 *μ*g of biotin-labeled RNA from each experimental sample.

### 2.5. Microarray Hybridization, Scanning, Normalization, and Annotation

Hybridization was carried out according the instructions supplies with the CodeLink Controls and Buffer Kit (Applied Microarrays, Inc.). For each experimental sample, 10 *μ*g of biotin-labeled material was the nominal amount of material used on the CodeLink Bioarrays. The arrays and labeled material were incubated together at a constant temperature of 37°C overnight. GenePix 4400A (Molecular Devices, Inc.) was used to scan each labeled array. CodeLink Expression Analysis v5.0 (Applied Microarrays, Inc.) software was used for the data analysis. Net intensity was calculated by withdrawing surrounding background of the spot from row intensity. Normalization is performed by adjusting the median of all the read microarray data with a fixed value. Microarray Data Analysis Tool version 3.2 (Filgen, Inc.) was used as described in the manufacturer's instruction for all subsequent data analysis. This software uses pathway information and data from either GenMAPP (http://www.genmapp.org/) or WikiPathways. We used data from the WikiPathways database because it is an open, collaborative platform dedicated to curating biological pathways (http://www.wikipathways.org/index.php/WikiPathways).

## 3. Results 

### 3.1. Translatome Analysis of Diarylheptanoid-Treated BJAB Cells

**Figure 3:**
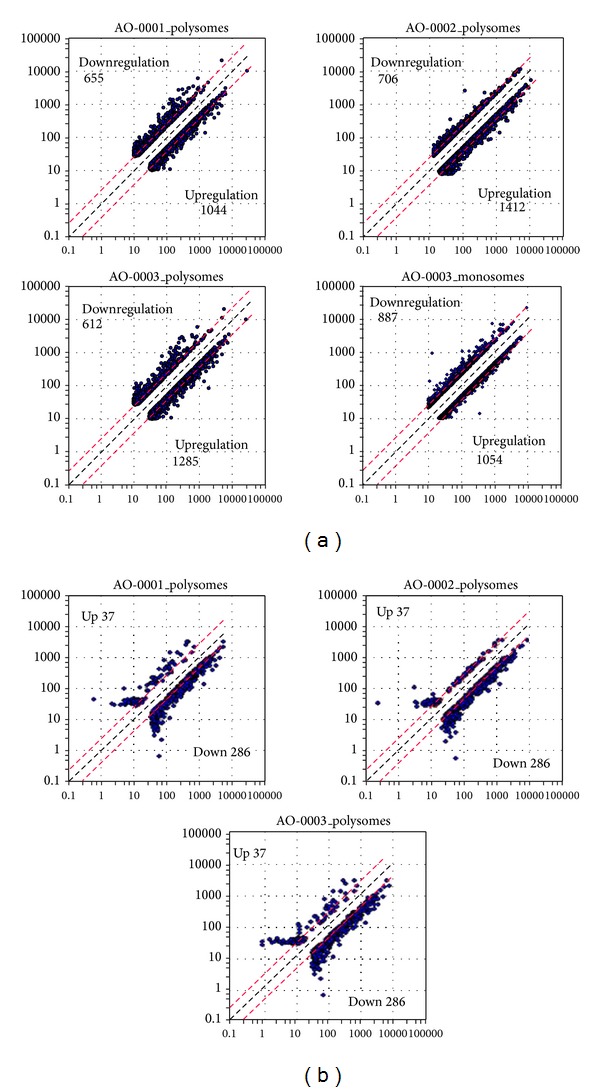
Scatter plot of normalized intensities versus control intensities (a) and of normalized intensities of genes affected by each diarylheptanoid (b). (a) Net intensity for each spot: the net intensity was calculated by subtracting surrounding background intensity, which was derived from row intensity, from the intensity of the respective spot. Normalization was performed by adjusting the median of all the read microarray data relative to a fixed value. After the net intensity of each probe was normalized, a sample versus control ratio was calculated. For those probes with 2-fold upregulation (Ratio ≥ 2) or downregulation (Ratio ≤ 0.5) in treated cells and that exhibited a normalized net intensity of more than 40, the net intensity values were plotted. (b) Normalized net intensity values of genes that were regulated by each diarylheptanoid were potted versus control normalized net intensity values.

To catalogue ribosome loading onto BJAB mRNAs in the presence or absence of diarylheptanoids isolated from* A. officinarum* (Figure 1), we generated polysomal profiles of BJAB cells under each of four conditions (Figure 2). Treatment with AO-0001 or AO-0003 decreased ribosome loading onto mRNAs in BJAB cells. We quantified the fraction of all mRNAs that bound more than 2 ribosomes; we then labeled these polysome-associated mRNAs with biotin and used the labeled mRNAs to label CodeLink Bioarrays. After normalizing net intensity for each probe, sample versus control ratios were calculated for each probe. Probes indicating more than 2-fold upregulation (ratio ≥ 2) or 2-fold downregulation (ratio ≤ 0.5) of the respective transcript or gene are listed in Table 1. The microarray analysis indicated that each of AO-0001, AO-0002, and AO-0003 altered (downregulated or upregulated) polysomal loading of more than 3,000 transcripts/genes (Table 1). Treatment versus control net intensity values were plotted for any transcript that exhibited a normalized net intensity value greater than 40 and that was upregulated (ratio ≥ 2) or downregulated (ratio ≤ 0.5) (Figure 3(a)). Total mRNA isolated from monosome fractions of AO-0003 treated BJAB cells was also used for DNA microarray analysis; these data were processed and are plotted in Figure 3(a). Each of AO-0001, AO-0002, and AO-0003 caused downregulation of 37 genes and upregulation of 286 genes in the treatment versus control normalized net intensity values which were plotted for each of these transcripts. The plots of AO-0001-affected transcripts and AO-0003-affected transcripts were very similar. Each of AO-0001 and AO-0002 caused downregulation of multiple genes encoding proinflammatory mediators [8, 20]; AO-0001 downregulated interleukin 8 (ratio of net intensity; 0.409), interleukin 18 (IL-18) (0.489), macrophage inflammatory protein-1*α* (0.241), and epidermal growth factor receptor (0.439); AO-0002 downregulated IL-18 (0.487) and macrophage inflammatory protein-1*α* (0.330). Each of AO-0001, AO-0002, and AO-0003 caused downregulation of the transcription factor notch-1 with the following net intensities, 0.244, 0.450, and 0.418, respectively.

### 3.2. WikiPathways Analysis

The Microarray Data Analysis Tool version 3.2 (Filgen, Inc.) was used for follow-up pathway analysis. This software uses pathway information that can be taken from either of GenMAPP (http://www.genmapp.org/) or WikiPathways. We chose to use the WikiPathways (http://www.wikipathways.org/index.php/WikiPathways) because it is publically available. AO-0001, AO-0002, and AO-0003 altered 7, 4, and 10 (resp.) of the pathways curated in WikiPathways database (Table 2). AO-0001 treatment of BJAB cells altered regulation of diurnally regulated genes with circadian orthologs. AO-0002 altered regulation of genes involved in proteasome degradation. AO-0003 altered regulation of genes involved in calcium regulation in the cardiac cell, cell cycle, mitogen-activated protein kinases (MAPK) signaling, or some combination thereof.

Notably, each diarylheptanoid altered class A rhodopsin-like GPCRs and the mRNA processing pathway (Table 2). Based on the WikiPathways analysis, AO-0001, AO-0002, and AO-0003 affected 35, 34, and 46 (resp.) mRNA processing-related genes; moreover, 16 of these transcripts were upregulated by all three diarylheptanoids.

### 3.3. Features of 5′ UTRs of Transcripts Regulated by Each Diarylheptanoid, AO-0001, AO-0002, and AO-0003

Different types of* cis*-acting elements encoded in mRNA 5′ untranslated region (5′ UTR) sequences can mediate regulation of translational initiation; such elements include (1) secondary structure that can block a scanning ribosome and thereby inhibit recognition of an AUG initiation codon, (2) IRESs that stimulate cap-independent translation, (3) protein binding sites that either repress or promote translation in response via transacting factors, and (4) upstream AUG codons, in some cases, associated with upstream open reading frames (uORFs) [21]. We analyzed in-frame uORF and terminator codons of the 5′UTR of 329 transcripts that were each regulated by each of the diarylheptanoids, AO-0001, AO-0002, and AO-0003 (Table 3). Of these transcripts, 49 (14%) contained a uORF, and uORFs were present in both downregulated and upregulated transcripts. Additionally, 53% of the upregulated transcripts each contained an in-frame terminator codons.

Interactions between RNA binding proteins and RNA elements control mRNA translation [22]. To date, at least 25 cellular IRES-containing mRNAs have been identified [23–28]. Among these cellular IRES-containing mRNAs, AO-0001 upregulated the mRNA encoding the insulin receptor (ratio of net intensity: 2.582), and AO-0002 downregulated the human inhibitor of apoptosis 2 protein transcript (0.448).

## 4. Discussion

Recently, Tebaldi et al. showed that the analysis of the translatome, an intermediate between the transcriptome and the proteome comprising polysome-associated mRNAs, can provide substantial and somewhat surprising information [17]. Translatome analyses can identify gene ontology (GO) terms within gene sets that represent the transcriptome, translatome, or proteome and then compare between sets with regard to GO term; this approach parses information about cellular components, molecular functions, and biological processes subject to differential regulation at different levels of gene expression [29]. Translatome analysis renders detection of “stress response” and “translation” related genes compared with transcriptome analysis. In this study, 16 transcripts that relate to mRNA processing were identified from the WikiPathways database (Table 2).

Each of AO-0001, AO-0002, and AO-0003 has anti-inflammatory effects and inhibits tumor progression [1, 2]. The dose (*μ*mol/ear) of 50% inhibitory dose of TPA-induced inflammation was AO-0001 < AO-0002 < AO-0003. Notably, the number of downregulated inflammatory related transcripts was greatest with AO-0001; it was fewer with AO-0002 and fewest with AO-0003. Future examination of the relationships between the anti-inflammatory effect of each diarylheptanoid and the resulting corresponding levels of IL-8, IL-18, macrophage inflammatory protein-1 *α*, notch-1, and epidermal growth factor in vivo is important.

AO-0001 has lower cytotoxic effects on IMR-32 cells than does AO-0003 [3]. AO-0003 had larger effects on the BJAB translatome than did AO-0002, which had a smaller effect at the same concentration (40 *μ*M); however, AO-0002 caused higher upregulation of genes encoding class A rhodopsin-like GPCRs, mRNA processing proteins, and proteasome-related proteins than did AO-0003 (Figure 2, Table 2). Each of AO-0001, AO-0002, and AO-0003 exhibits antimeasles virus activity; however, AO-0001 and AO-0003 do not exhibit anti-respiratory syncytial virus (-RSV) activity [11]. It is possible that some host factors such as splicing factors or heterogeneous nuclear ribonucleoproteins (hnRNPs) listed in Table 2 might affect virus structure and/or replication cycle. Among the proteins encoded by these transcripts, heterogeneous nuclear ribonucleoprotein C (C1/C2), heterogeneous nuclear ribonucleoprotein K, human 55 kDa nuclear matrix protein/octamer-binding non-POU domain containing, and PTB 1 are each identified as an IRES-transacting factor [21]. Overall, our findings provided new insights into the mode of action of diarylheptanoids from* A. officinarum* with regard to anti-inflammatory, antitumor promotion, and antiviral effects.

## 5. Conclusions

This translatome analysis identified genes that were upregulated or downregulated by 2 h exposure to each of three diarylheptanoids from* A. officinarum*. Notably, genes related to mRNA processing and class A rhodopsin-like GPCRs were upregulated or downregulated by each of the three diarylheptanoids. Based on these findings, we propose that the biological effects of* A. officinarum* diarylheptanoids are mediated via control of expression of specific genes. Translatome analysis might be useful for advancing our understanding of molecular effects of complementary and alternative medicines.

## Figures and Tables

**Table 1:** Number of transcripts exhibiting altered polysomal loading in BJAB cells following treated with AO-0001, AO-0002, or AO-0003.

	AO-0001		AO-0002		AO-0003		Common Gene
	Probe∗	Gene	Probe∗	Gene	Probe∗	Gene	
Downregulated (ratio ≦ 0.5)	965	634	950	663	900	585	37
Upregulated (ratio ≧ 2)	1308	994	1960	1349	1574	1222	286
Total altered	**2273 **	**1628**	**2910 **	**2012**	**2474 **	**1807**	**323**

*Total of 54359 probes were used.

**Table 2:** Number of WikiPathways transcripts that exhibited altered polysomal loading following AO-0001, AO-0002, or AO-0003.

WikiPathways	Total gene no.	AO-0001		AO-0002		AO-0003	
Pathway name		*z* score	Altered gene number	*z* score	Altered gene number	*z* score	Altered gene number
Alanine and aspartate metabolism WP106 41117	12	2.73	4			3.33	5
Apoptosis WP254 41184	79	−2.18	2			−2.45	2
Calcium regulation in the cardiac cell WP536 41204	145					−2.19	8
Cell cycle WP179 45137	72					2.93	16
Diurnally regulated genes with circadian orthologs WP410 41104	42	2.52	9				
DNA replication WP466 41036^∗1^	38	3.95	11				13
Glycolysis and gluconeogenesis WP534 41077	45	3.29	11				14
GPCRs, class A rhodopsin-like WP455 41121^∗2^	227	−3.40	7	−2.67	13	−3.05	11
MAPK signaling pathway WP382 41048^∗3^	153					−2.10	9
mRNA processing WP411 45374^∗4^	110	7.40	34	5.54	31	9.80	45
Proteasome degradation WP183 45274	52			3.09	13		
Translation factors WP107 41026	42			3.02	11	3.05	11

WikiPathways transcripts with a *P* value < 0.5 are listed.

^∗1^DNA: deoxyribonucleic acids

^∗2^GPCRs: G protein-coupled receptors

^∗3^MAPK: mitogen-activated protein kinases

^∗4^The following 16 transcripts were each upregulated after treatment with each diarylheptanoid (AO-0001, AO-0002, or AO-0003). CSTF1: cleavage stimulation factor, 3′ pre-RNA, subunit 1, 50 kDa; CSTF3: cleavage stimulation factor, 3′ pre-RNA, subunit 3, 77 kDa; DDX1: DEAD (Asp-Glu-Ala-Asp) box helicase 1; FUS: fused in sarcoma; HNRNPAB: heterogeneous nuclear ribonucleoprotein A/B; HNRNPC: heterogeneous nuclear ribonucleoprotein C (C1/C2); HNRNPK: heterogeneous nuclear ribonucleoprotein K; NONO: non-POU domain containing, octamer-binding; PTBP1: polypyrimidine tract binding protein 1; PRPF40A: PRP40 pre-mRNA processing factor 40 homolog A (S. cerevisiae); PRPF6: PRP6 pre-mRNA processing factor 6 homolog (*S. cerevisiae*); SRSF7: serine/arginine-rich splicing factor 7; SNRNP70: small nuclear ribonucleoprotein 70 kDa (U1); SF3A3: splicing factor 3a, subunit 3, 60 kDa; SF3B2: splicing factor 3b, subunit 2, 145 kDa; U2AF2: U2 small nuclear RNA auxiliary factor 2.

**Table 3:** Features of 5′ UTRs in transcripts for which AO-0001, AO-0002, and AO-0003 each caused altered polysomal loading.

	Upstream open reading frame		In-frame stop codon		Other features	
	Number of transcripts	% of total	Number of transcripts	% of total	Number of transcripts	% of total
Downregulated (Ratio ≦ 0.5)	5	14%	16	43%	16	43%
Upregulated (Ratio ≧ 2)	41	14%	156	53%	95	33%

## Abbreviations

5′ UTR: 5′ untranslated region
BJAB: Human B-lymphoblastoma cell line
DMSO: Dimethyl sulfoxide
DNA: Deoxyribonucleic acids
GO: Gene ontology
GPCRs: G protein-coupled receptors
hnRNPs: Heterogeneous nuclear ribonucleoproteins
IL-18: Interleukin 18
IRES: Internal ribosome entry site
LSB: Low salt buffer
MAPK: Mitogen-activated protein kinases
mRNA: Messenger ribonucleic acid
RPMI medium: Roswell Park Memorial Institute medium
RSV: Respiratory syncytial virus
TPA: 12-O-Tetradecanoylphorbol-13-acetate
uORF: Upstream open reading frame.
